# What Is Homœopathic Prescribing?

**Published:** 1884-06

**Authors:** P. P. Wells


### HOMŒOPATHIC PHYSICIAN,

A MONTHLY JOURNAL OF MEDICAL SCIENCE.

"If our school ever gives up the strict inductive method of Hahnemann, we are lost, and deserve only to be mentioned as a caricature in the history of medicine."—CONSTANTINE HERING.

Vol. IV. JUNE, 1884. No. 6.

### WHAT IS HOMŒOPATHIC PRESCRIBING?

Something more is necessary to this than styling one's self a homœopathic physician; something more than correct diagnosis; something more than minute doses of medicine; something more than to have graduated from a college, the adjective nominal of which is "Homœopathic;" something more, it may chance, than an honest endeavor to carry out in clinical duties instructions which the practitioner may have there received; something more than honesty of purpose, though this may well be supposed to be a constituent in every true homœopathic prescription; something more than all these combined is necessary to an answer to this question, though these may all, more or less, enter into it.

The question has been suggested by reading a report of an unsuccessfully treated case in the *U. S. Medical Investigator* of Feb. 16th, 1884. The case ought to be instructive. And here we may remark, we wish more practitioners would report unsuccessful cases, if they have them, and who, at times, has not? They are often more illustrative of principles than are reports of cures. In this respect the case with which we now propose to deal is an eminent example, as it is also of the evident honesty and sincerity of the reporter. He had also the courage to report his want of success, which we would heartily applaud, while we remark that, as compared with reports of cases which seem inspired by a desire to display the skill of the prescriber, this, of a failure, is much to be preferred. He also has the frankness to say, "Please, readers of the *Investigator*, criticise me *severely*, and comment on treatment," etc. This is what we now propose to do, acknowledging ourselves disarmed of all disposition to "severity" by the evident earnestness and humility of the request.

The first remark we make on this report is, that the first requisite of *homœopathic* treatment of the sick is presumably absent from it. We refer to the "*totality of the symptoms.*" The symptoms given as precedent of the first prescription are so few and of such a character, we can hardly suppose there were not others coexistent with them not here given. We are, of course, ignorant of these, and of how important an influence they should have had in controlling this *first prescription*. Here is the statement of them :

"Jan. 20th I was called on to prescribe for *vomiting* and *intense* headache. I gave Ipecac 3x. and Atropia 3 (in water.)* At night was called, 12 M., to attend. She vomits and vomits. I found the pupils largely dilated, tongue furred, pulse rapid and small."

"Vomiting and intense headache"—and this is all. From this brief statement we are warranted in saying, more than a Hering or a Hahnemann would be required to say of any drug, *this* is the specific cure for the child (about nine or ten years old). Scores of drugs are recorded as having caused vomiting and headache, and unless we can have the *kind* of vomiting and *kind* of headache, as disclosed by accompanying symptoms, no intelligence can decide which of them is the specific for a case only so characterized. And in the absence of these necessary symptoms, a mere diagnosis, now so much thought of (but not too much), and so rightfully insisted on from the prescriber, is equally impossible.

The first prescription in the case is notable as describing the state of mind of the reporter, as before our law, as he understood it, viz. : *Vomiting—Ipecac ; Headache—Atropia*. Two facts—two medicines, and a failure; whereas, the "totality," if brought to light and acted on, might have led to one, the specific, and therefore to a cure.

But there is another remarkable fact which followed the use of what some will certainly regard as unnecessarily large doses of the drugs given. Ipecac is followed, as stated in the report,

* Was this also 3d decimal? The report does not say.

thus:—"She vomits and vomits." Evidently here was an aggravation of this element of the case from Ipecac. And the result of the Atropia was not less remarkable or less threatening a fatal result, as shown in the new symptoms already developed at midnight—"pupils largely dilated, tongue furred, pulse rapid and small." Here was another development of medicinal symptoms which, like the increase of vomiting, failed to enlighten the medical attendant, either as to the nature of these increased sufferings, or the inordinate magnitude of the doses given, as appears by the report, which goes on to say—

"Gave Aconite 1x and Belladona 1x. Jan. 21st.—No better. A. M.—Gave Ipecac 6x, two hours. P. M.—No better. Gave wine of Antimony, two drops every half hour. Vomiting stopped. Jan. 22d, 12 M.—Vomiting returned; nothing can be retained on the stomach, intense thirst, all comes up, is restless. Wine stopped. Gave Canth. 1x and Apis. mel. 2x.*

Why Aconite and Belladonna? If the law of similars were his guide in selecting his remedies, and he were possessed of only a moderate share of knowledge of materia medica, he must have known that if the law called for either of these, it could not for the other. Law calls for the *most* like, and *both* could not be this, not being in their effects identical. Then, certainly, one of them was wrong, and, therefore, being a drug, *i. e.*, a *sick-making* substance, could only be injurious. So it was that Jan. 22d the patient was no better. The vomiting, which had been temporarily checked by Antimony, returned at noon,† and now the patient got Cantharis 30, and, being no better, in the afternoon got Canth. 1x and Apis. 2x.

We can see in the report no call for either of these drugs by the law. Cantharis is certainly not suggested by any facts of the case yet given, and we are left wholly in the dark as to the motive for its selection. Presumably there was no good reason, as no relief to the patient followed its use. The report goes on:

---

*No reported case which has come to my knowledge has had in it so many points illustrative of our needs of the first cardinal corollary of our basic law —the "*totality of the symptoms.*" This is our *chart*, by which, and by which alone, we are to be guided in sailing our disabled charge into the port of health, our law of similars being our compass. Now, if the mariner's chart has but one point expressed on its surface, how can it be a guide to his destined haven? His compass may be perfect and his ship stanch, but what are these to his safety or success if this be the character of his chart? So it is in the absence of our therapeutic chart, and we are given up, as in the case before us, to go on *one* symptom. In such a case, to use a common expression, "*we are all at sea.*" Then, therapeutically, we don't know where we are.

† Was this the result of the continued use of the Antimony, after the vomiting had stopped?

"January 20th.—No action from the bowels for three days,* tongue now clearing off, but is turning *brown* with red edges. Thirst almost gone; gave an injection, * * has eaten nothing since January 15th to amount to anything. Nux v. 1x, one dose, then Apis mel. and Rhus tox."

For evidences of the medicinal effects of the first prescription, which in each succeeding report are so apparent, see Allen's *Encyclopædia*, Vol. I, article Atropinum. Up to this date it is certainly a question whether the sufferings from inordinate doses of drugs were not greater than from any remainder of the natural disease. And now, as if to make recovery impossible, the patient gets three different drugs on the same day, neither of them, so far as can be seen from the report, in any degree promising relief to the patient by reason of similarity. These doses were also inordinately large, and this to an extent which would certainly in a susceptible patient preclude the possibility of any re-action in the direction of recovery. They could have very little curative relation to the case, as neither of them are, so far as we know, antidotal to Atropin, from which the patient was all this time suffering so greatly, as well as from the added impetus to these sufferings given by the Bell. immediately after it. And so it happened, just as should have been expected, the report says:

"January 24th.—No better; vomiting, some thirst, restless. Aconite (Bœricke's) 1x, *and* Rhus tox. P. M.—More restless. Delirious at times Counsel from Ottumwa. He gave Ipec. 3x, Bry. 2x, Rhus 2x.† January 25th.—No better. Delirious. Strikes, bites. (Atropine.) Tries to change bed."

Why these three drugs again? Each had failed to benefit the patient, except Bry. and there was no apparent good reason for expecting any better result from adding this inappropriate one to the series of previous failures. Neither of them met the evils of the drug sickness which had followed previous ones and wrong dosing. But now was added to these symptoms of Rhus.

"Gave Hellebore tincture four gtts. in three ounces of water, teaspoonful each hour. P. M.—No better. Gave Hyoscyamus tincture in the same way. January 26th.—Better. Gave Glycerine one and a half tablespoonfuls. Bowels moved in three-quarters of an hour. Hyoscyamus as before every two hours. January 27th.—Worse. Again Bry. and Rhus. Prognosis, fatal."‡

---

* The reporter was evidently not wholly free from the old-school superstition of "moving the bowels," which makes so large a part of the therapeutics of that school, however he may have found deliverance from other practical trash from the same source, by his homœopathic education and experience.

† The counsel evidently believed in the *shot-gun practice*.

‡ Will the reporter remember his patient was no better after this departure from homœopathic practice—"moving the bowels"? That she was immediately after worse.?

It can hardly be surprising that such should have been the case. Young human nature of no more than average toughness could hardly be expected to survive such a series of such dosings. A very hardy specimen might perhaps occasionally overcome them, but to expect this from average constitutions would often be exposure to unpleasant disappointments. In the report of the 26th, which gave the first opportunity to say better, it is, from the homœopathic standpoint, fair to inquire—why not, then, leave the Hyoscyamus to work itself out? This would have been according to law and correct practice. Why interfere with its action further by giving Glycerine? How much had this abomination to do with the next report—*worse?* This and continued large doses of Hyoscyamus no doubt were responsible for this. The thought would seem to have been, the more of a remedy which has relieved the better, and the usual result of overdosing, as in case of the other drugs, was realized. She was so much better she recognized her friends. She got continued doses of Hyoscyamus and the consequence was:

January 27th.—Worse. Again got Bry. and Rhus [both of which had been tried and had done no good]. * * * Wanted to be discharged. Would not let me off. January 28th, 29th, and 30th.—About the same. *Convulsions* were arrested by Hyoscyamus, etc."

What made the patient worse on the 27th? What, indeed, but the continuance of the drug which should have been suspended when amendment followed its use, so long as that amendment continued. And what caused the convulsions? Did they have their origin in the fearful Atropin? This may well be, and in the absence of any other fact which can stand accepted as their cause we are left to this, which, more than likely, was the origin of this distressing symptom.

"The patient got Colch. cc., and became rapidly worse. February 1st.—Back again to Rhus tincture, and now I gave Aconite obtained from Europe (German tincture from Dresden); fever dropped off on its exhibition. Patient now is drowsy, tongue clean, *eyes protruding.* Gave Opium 6x. February 2d.—No better. Gave cathartic. It came up in ten minutes. Nux v. cc. No relief. Atropia 1x. Patient become more straight, eyes become more natural. February 3d.—Worse, pulse slower, patient comatose, but is aroused by speaking frequently. Rhus tox. 3, Op. 6."

February 4th the reporter was discharged, and his successor was an old allopathic doctor, who saw through the case at once, and found "all she needed" was "*a good physicking,*" and then "she would get along." She got the "physicking." She got two of them, and then on the afternoon of the 5th she "got along" out of her sufferings, and out from this world!

The reporter asks for a diagnosis of this case from the "readers of the *Investigator*." With only the report (the substance of which we have given) before us, we should say the diagnosis of the original attack is impossible. The mixed character of the case, as it progressed, became sufficiently apparent. The reporter seems to feel a degree of relief from the thought that he is "not legally bound to certify to her death." This is easy to be understood. If it were our duty to render this certification, truth would compel us to say—died from *x*, *y* or *z*, or some other unknown quantity, aided by sundry drugs taken in inordinate doses, but whether these or the original disease is to be credited with the largest share of the destructive agency is to this certifyer unknown.

We remark on the practice of this case, that whatever the public or the reporter may style the professional status of this doctor, this report is utterly destitute of evidence that any one prescription in the progress of the treatment was in any particular homœopathic to the case of the sick child. If in reply to this it be asked—Is not Ipecac homœopathic to vomiting? not necessarily, we reply, and not at all in a vast number of cases. It is only homœopathic to vomiting of a certain character attended by concomitants like to effects of this drug taken by healthy persons. In this case there is no evidence of this character of the vomiting, nor of the existence of these concomitants. And then—Is not Belladonna homœopathic to headache? We reply, only to its *own kind* of headache; and this *kind* is determined as in the vomiting, by its own characteristics and the concomitants attending it. These concomitants, by the way, are often the most important indices to the specific cure. The importance, then, of a knowledge of the "*totality of the symptoms*," as writers often, and the law always, declare indispensable to a true specific prescription, becomes apparent, because in this "*totality*" is contained these indispensable concomitants. And then, *if* Belladonna or its alkaloid element were really homœopathic to the headache, or to the state of the brain revealed in part by this pain, then by reason of the similar action of the drug to this action of the life forces as affected by the morbid cause, the result of the inordinate doses of these drugs given could hardly fail of being fatal. The action of the doses being *like* that of the morbid cause, both in character and direction, it could hardly have been otherwise than that the influence of the artificial cause on the present, acting natural cause, should by this supplement of its action have carried it beyond the line of possible recuperation. Something of this seems apparent in this case by reason of the ever-recurring symptoms of Atropine given at the first visit.

Then there appears through the case a constant thought that the benefit of the dose was to be increased by increase of its magnitude, and if a medicine had failed of the expected results, the quantity of the dose was increased. This idea is *old school,* and with it Homœopathy has no affiliation. The benefit of *homœopathic* doses is only increased by a more perfect likeness, and not by increase of quantity at all. Increase of quantity, as we cannot but suspect in this case, may have much to do with hastening and insuring fatal results. And these, it may not be out of the way to remark, are altogether too large a price to pay for the sham heroic affected by some, who seem to give large and frequent doses to the sick to show they *are not afraid of them.** If they are not, their patients have the strongest reasons for being so. These and their doctors ought to know that these poisonous doses of drugs are not necessary to any cure, and, more than this, that smaller and innocuous doses cure much better, *i. e.,* more speedily, safely, and in much larger proportion. Doctors ought to know that these large and poisonous doses are never necessary and seldom useful, and homœopathic doctors, when resorting to them, that they have gone beyond the realm of genuine homœopathic practice, and true Homœopathy will not be held responsible for the results of this resort to old-school ideas and means by whomsoever practiced.

If we recur to the question at the head of this paper, with a view to its answer, we say homœopathic prescribing is ever and

---

*It happened many years ago that a patient of the writer passed from his care to that of a doctor who had, by the importunity of a friend of the patient, been called as a consulting attendant, the case being one of incipient phthisis. Some three days after the Doctor had assumed charge of the case I accidentally met him and inquired for the present condition of the patient. After answering as to this, he gave me the prescription he had left but a few minutes before. It was tincture of Ipecac, one teaspoonful in a wineglassful of water, a teaspoonful of which was to be taken every two hours. He gave me this gratuitous piece of information with an air of bravado, which said as plain as words could that he was not afraid. The second night after this my bell rang violently, and I was requested to go to the young lady as "quick as possible." When I reached the house the patient was dead. A gushing and copious hæmorrhage had saved the poor girl the experience of lingering suffering, through which phthisical patients usually come to their end. This, if it were a good, was all that resulted from this practice. The Doctor, most likely, was not instructed by this outcome of his rashness. But soon after this he found out somehow, that Homœopathy wouldn't do, and he went over to the old school, not being able to practice a successful Homœopathy, where he is still a shining light of the sort sometimes described as a *lucus a non.*

only solving the problem of the specific curative for each individual case of sickness as it is presented for treatment. It is always a *practical* fact, and not necessarily, in any degree, a matter of theory. Whatever of theories may be entertained as to the nature of the sick-making power, its mode of action, or of the results following, or of the nature of the curing force, its mode of action in effecting the cure, or of the sum of it required for the speediest and best accomplishment of this, the fact remains that for the discovery of this force we have to do with plain, simple facts, and not with theories at all, and any mixing or attempting to mix whatever of theories with the search for the specific is only a detriment to all best interests involved, and an active contributor to probable disappointment. The search for the specific (homœopathic prescribing) is always a simple affair, *i. e.*, simple as opposed to complex. It always, and in all cases, deals with plain, *known* facts, and excludes all of the nature of guessing. On the one side the facts of the sickness (the *totality* of the symptoms, to which we have so often to recur, and which sometimes are so difficult to draw out), and on the other, to find in the facts of the materia medica the most exact likeness to those of the sickness, which, when found, the whole duty of the homœopathic prescriber is done, when he has given the drug so found, and managed its use according to the wise directions of the great master. This is homœopathic prescribing, and nothing else is. Any attempt to mix with this any other science, no matter of what value, is only a damage to our great, simple, and beautiful science of therapeutics. It needs no help whatever from other sciences outside itself. God made this science, the practical application of which we have attempted to describe, in itself, equal to the needs of all healers. What is most like? Find it and give it, and this *is homœopathic prescribing*—which that in the report we have reviewed certainly was *not*.

